# Dancing Electrohydrodynamic Tip Streaming Modulated by Faraday Instability

**DOI:** 10.1002/advs.202508649

**Published:** 2025-10-14

**Authors:** Qiyou Liu, Bingqiang Ji, Yafeng Zou, Dingwei Zhang, Hu Sun, Qingfei Fu, Lijun Yang

**Affiliations:** ^1^ School of Astronautics Beihang University Beijing 100191 P. R. China; ^2^ State Key Laboratory of High‐Efficiency Reusable Aerospace Transportation Technology Beijing 100191 P. R. China; ^3^ National Key Laboratory of Aerospace Liquid Propulsion Xi'an 710100 P. R. China

**Keywords:** electrohydrodynamic printing, electrospray, faraday waves, instability, tip streaming

## Abstract

Tip streaming enables flow transitions from the millimeter scale to the microscale and even nanoscale, generating tiny droplets with broad applications in precision 3D printing, nanomaterial fabrication, and drug delivery. In these applications, high‐frequency jetting is required to improve efficiency. However, understanding and controlling high‐frequency tip streaming remain challenging. Here, a series of dancing electrohydrodynamic (EHD) tip streaming phenomena is reported, characterized by subharmonic ejection modes under high‐frequency electric fields. The underlying mechanism of this intriguing phenomenon is elucidated, which stems from global meniscus oscillations induced by Faraday instability, followed by jetting at the Faraday wave crests due to local interfacial instability. The optimal excitation frequencies of these ejections are governed by the natural frequencies of Faraday instability, while a maximum electric Bond number comparing electric and capillary effects determines the ejection voltage threshold, thus enabling precise control of high‐frequency EHD tip streaming. Owing to the high excitation frequency and multi‐directional jetting feature, the dancing EHD tip streaming demonstrates enhanced throughput and multi‐path delivery, opening new and exciting prospects for various drop‐on‐demand technologies.

## Graphical abstract

A novel dancing electrospray phenomenon is reported characterized by high‐frequency, robust, and multi‐directional alternating ejection modes. This phenomenon arises from the electrohydrodynamic (EHD) tip streaming at the crests of Faraday waves excited on the liquid meniscus, and it offers significant advantages in high‐throughput and multi‐path delivery for applications such as EHD printing and electrospinning.

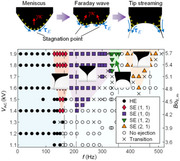

## 1 Introduction

Tip streaming is a distinctive interfacial flow featured by a thin fluid stream emitted from the tip of a conical‐shaped liquid interface.^[^ ^1^, ^2^, ^3^ ^]^ It is driven by shear stress from the outer stretching flow or interfacial stress from the electrohydrodynamic (EHD) effect, which overcomes surface tension and causes local interfacial instability, leading to the formation of a singular cusp and associated ejections.^[^ ^4^, ^5^, ^6^, ^7^ ^]^ The EHD tip streaming, a prominent example referred as electrospray, can produce tiny ejections as small as sub‐micron or even nanometer scale due to its intrinsic singularity.^[^ ^8^, ^9^ ^]^ It has broad applications in nanomaterial production,^[^ ^10^, ^11^, ^12^ ^]^ mass spectrometry,^[^ ^13^, ^14^, ^15^ ^]^ drug delivery and packaging,^[^ ^16^, ^17^ ^]^ electrospinning,^[^ ^18^, ^19^ ^]^ and EHD printing.^[^ ^20^, ^21^, ^22^ ^]^ Over the past century, extensive efforts have been made to explore the complex electrohydrodynamics behind the electrospray. However, due to the inherent complexity and strong parametric dependence of the electrospray, its precise control has long been a challenge. Even under a steady electric field, electrospray is modulated by plenty of parameters, such as liquid properties and flow rate, and the system geometry, yielding various ejection behaviors with a strong multiscale nature.^[^ ^23^, ^24^ ^]^ Therefore, understanding and controlling the ejection in electrospray is of both fundamental and technical significance.

Under a steady electric field, the scaling laws and stability of EHD tip streaming have been extensively studied.^[^ ^25^, ^26^ ^]^ However, oscillating electric fields offer the advantage of actively controlling the onset of tip streaming, making this approach a promising strategy for drop‐on‐demand printing in manufacturing applications.^[^ ^27^, ^28^ ^]^ Using voltage waveforms such as pulses or sine waves, the ejection frequency and droplet size can be individually controlled by adjusting the applied voltage amplitude and frequency.^[^ ^29^, ^30^, ^31^, ^32^ ^]^ In practice, the pulsating Taylor‐cone jet mode is usually applied since the ejection is harmonic, axisymmetric, and thus easy to control.^[^ ^33^, ^34^ ^]^ However, this ideal controlled ejection behavior is only effective at low frequencies, where the cone‐jet forms and vanishes within each voltage cycle. At higher frequencies, the ejection can no longer keep pace with the driving signal, as the ejection frequency significantly lags behind the applied voltage frequency, and resonant meniscus oscillations may emerge at specific frequency ranges.^[^ ^35^, ^36^, ^37^ ^]^ The physical nature underlying the complex electrospray phenomena in oscillating electric fields remains elusive, which poses a critical barrier to the broader application of electrospray in on‐demand ejection technologies. Therefore, the motivation of this study is to investigate the dynamics of EHD tip streaming under periodic electric fields and provide guidance for achieving controllable tip streaming.

In this work, we report a novel dancing EHD tip streaming phenomenon characterized by a series of subharmonic ejection modes under high‐frequency electric fields. Unlike the classic harmonic Taylor cone‐jet mode, these ejections occur at the crests of Faraday waves induced by oscillating electric fields, exhibiting higher ejection frequencies and multi‐directional jetting behavior. We further unravel how the Faraday instability modulates the tip streaming behavior and establish control strategies for different ejection modes regarding the frequency and amplitude of the applied voltage. Distinct from previous studies, our strategy focuses on the global stability of the meniscus, utilizing Faraday waves to modulate tip streaming through the coupling between interfacial oscillations and local ejection dynamics. The dancing EHD tip streaming demonstrates a high droplet throughput rate and the capacity of multi‐path delivery, opening new avenues for various applications such as drop‐on‐demand printing and electrospinning.

## 2 Results

### 2.1 Dancing EHD Tip Streaming Experiments

**Figure 1:**
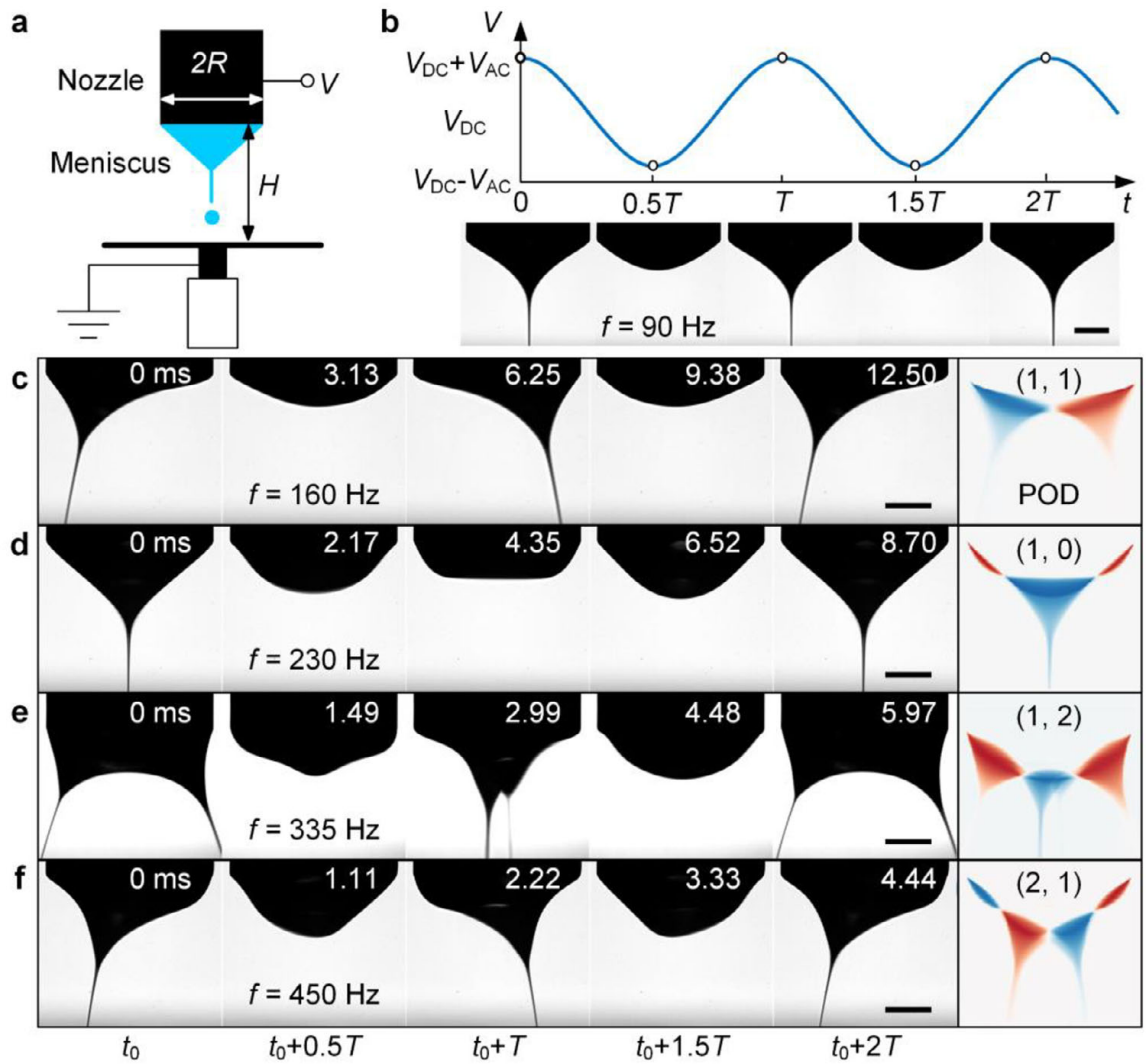
Dancing EHD tip streaming experiments. a) Sketch of the experimental setup. The nozzle has an outer radius of *R*, and the distance between the nozzle and the grounded electrode is *H*. The liquid is supplied with a syringe pump. b) The voltage *V* with a period *T* applied to the nozzle and the corresponding harmonic pulsating Taylor‐cone jets at a low frequency (*f* = 1/*T*) of 90 Hz. Experimental snapshots and POD analyses of different dancing EHD tip streaming modes with *V*_DC_ = 1.9 kV, *V*_AC_ = 1.8 kV, and *Q* = 20 µL min^−1^ at c) *f* = 160 Hz, d) 230 Hz, e) 335 Hz, and f) 450 Hz. POD analysis of experimental images reveals the dominant ejection modes. Scale bars represent 0.5 mm.

We conduct the electrospray experiments under an oscillating electric field, which is provided by a horizontal grounded electrode plate and a vertical metal nozzle connecting to a signal generator (**Figures** **1**a; S1, Supporting Information), with a voltage signal of *V* = *V*_DC_ + *V*_AC_ cos(2π *f t*) (Figure 1b). Here, *V*_DC_ represents the direct current (DC) offset voltage, while *V*_AC_ and *f* denote the amplitude and frequency of the alternating current (AC) voltage, respectively. The nozzle has an outer radius of *R* = 0.9 mm, which sets the perimeter of the liquid meniscus, and the distance between the nozzle and the electrode plate is *H* = 2.0 mm. We adopted 1‐octanol, a widely employed leaky dielectric liquid,^[^ ^38^, ^39^ ^]^ as the working fluid, supplied by a syringe pump with a stable flow rate *Q*. Motion of the meniscus and EHD jets is recorded using a high‐speed camera (Photron, Fastcam Nova S16). *V*_DC_ is set high enough to stretch the meniscus but remains below the threshold required to trigger EHD tip streaming.^[^ ^25^, ^40^ ^]^ By superimposing a sufficiently large *V*_AC_, pulsed EHD jetting is triggered. At a relatively low frequency, the liquid meniscus oscillates axisymmetrically, producing periodic jetting at the tip of the curved meniscus (Figure 1b; Movie S1, Supporting Information). This jetting occurs at the voltage peak in each period (*T* = 1/ *f*), which is known as the classic pulsating Taylor‐cone jet.^[^ ^33^, ^41^ ^]^

However, with increasing AC frequency, we observed a series of well‐organized and distinctive ejections. The liquid surface also exhibited a pronounced conical shape, characteristic of transient tip streaming.^[^ ^3^ ^]^ Different from the classic pulsating cone jet with a single crest at the axis, the dancing EHD tip streaming exhibits distinct spatial characteristics along the radial and azimuthal directions at the meniscus. Through proper orthogonal decomposition (POD) analysis of the high‐speed camera images,^[^ ^42^, ^43^ ^]^ we categorize the modes of the dancing EHD tip streaming using pairs of numbers (*n*, *m*), where *n* denotes the number of radial nodes and *m* denotes the azimuthal wavenumber (Text S2, Supporting Information). Four dancing modes, (1, 1), (1, 0), (1, 2), and (2, 1), are identified. Specifically, the (1, 1) mode is characterized by alternating jets on opposite sides (Figure 1c; Movie S2, Supporting Information), the (1, 0) mode is axisymmetric (Figure 1d; Movie S3, Supporting Information), and the (1, 2) mode exhibits jets simultaneously emitted in two opposing directions (Figure 1e; Movie S4, Supporting Information). The (2, 1) mode features alternating jets on two sides, with an additional smaller crest on the meniscus opposite the larger one. This smaller crest does not produce EHD jets, as EHD tip streaming tends to occur at peaks with the highest curvature (Figure 1f; Movie S5, Supporting Information). The jet diameter was measured to be ≈ 30 µm (Figure S2, Supporting Information). All these dancing EHD tip streaming modes are found to be periodic and sustainable at a constant flow rate. The long‐term stability of this phenomenon was verified by its ability to preserve excellent periodicity and robustness (Figure S3, Supporting Information). More generally, the dancing EHD tip streaming phenomenon has been validated in 1‐octanol, ethanol (Figure S6, Supporting Information), a polymer solution (Figure S7, Supporting Information), and a silver nanoparticle ink (Section 2.4), demonstrating its extensibility across liquids with distinct physical properties.

### 2.2 Mechanisms Underlying Different Ejection Modes

**Figure 2:**
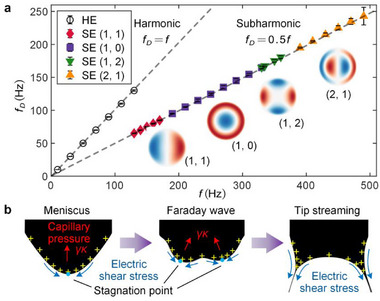
Mechanism and regulation of the dancing EHD tip streaming. a) Frequency response of the dancing EHD tip streaming to the applied voltage. Here, *f* denotes the applied voltage frequency, and *f_D_* represents the frequency of the observed phenomenon. Experimental conditions: *R* = 0.9 mm, *V*_DC_ = 1.9 kV, *Q* = 20 µL min^−1^. The error bars indicate the standard deviation. Insets are the corresponding Faraday wave patterns obtained numerically. b) Schematic of the mechanism. Faraday waves on the meniscus shift stagnation points, where surface charges are enriched at the wave tips and yield a high electric shear stress overwhelming the surface tension, triggering the local interfacial instability for EHD dancing tip streaming.

The frequency response of the EHD tip streaming to the applied voltage across various modes is illustrated in **Figure** **2**a. At a low applied voltage frequency, the ejection frequencies of the conventional pulsating EHD tip streaming, *f_D_*, well match *f*, indicating a harmonic ejection (HE) mode, as reported by previous studies.^[^ ^25^, ^33^ ^]^ However, at an elevated *f*, the dancing EHD tip streaming repeats at a frequency *f_D_* = *f* /2, exhibiting subharmonic ejection (SE) modes. The subharmonic nature and spatially regular patterns of the dancing EHD tip streaming are reminiscent of the surface Faraday waves.^[^ ^44^, ^45^ ^]^ The oscillating electric field can excite Faraday waves on the liquid meniscus, where the curvature stands out at the wave crests, forming tips with high charge density,^[^ ^46^ ^]^ as shown in Figure 2b. The resulting localized electric shear stress overcomes surface tension, destabilizing the stagnation point and driving the tip streaming.^[^ ^6^, ^8^ ^]^ Acting as a global modulation on the meniscus, the Faraday waves shift the stagnation point from the center to the crests, where the localized tip streaming occurs. This illustrates a global–local instability coupling, in which the Faraday instability regulates the onset of tip streaming. In fact, the tip streaming is an intrinsically transient flow that can be sustained by an appropriate flow rate *Q*, as it leads to the loss of mass and charge from the precursor fluid shape.^[^ ^3^ ^]^ When we shut off the liquid supply (*Q* = 0), Faraday waves with the exact same patterns remain on the meniscus while tip streaming is killed, evidencing that the origin of the dancing EHD ejections is Faraday instability (Text S1 and Figure S4, Supporting Information).

**Figure 3:**
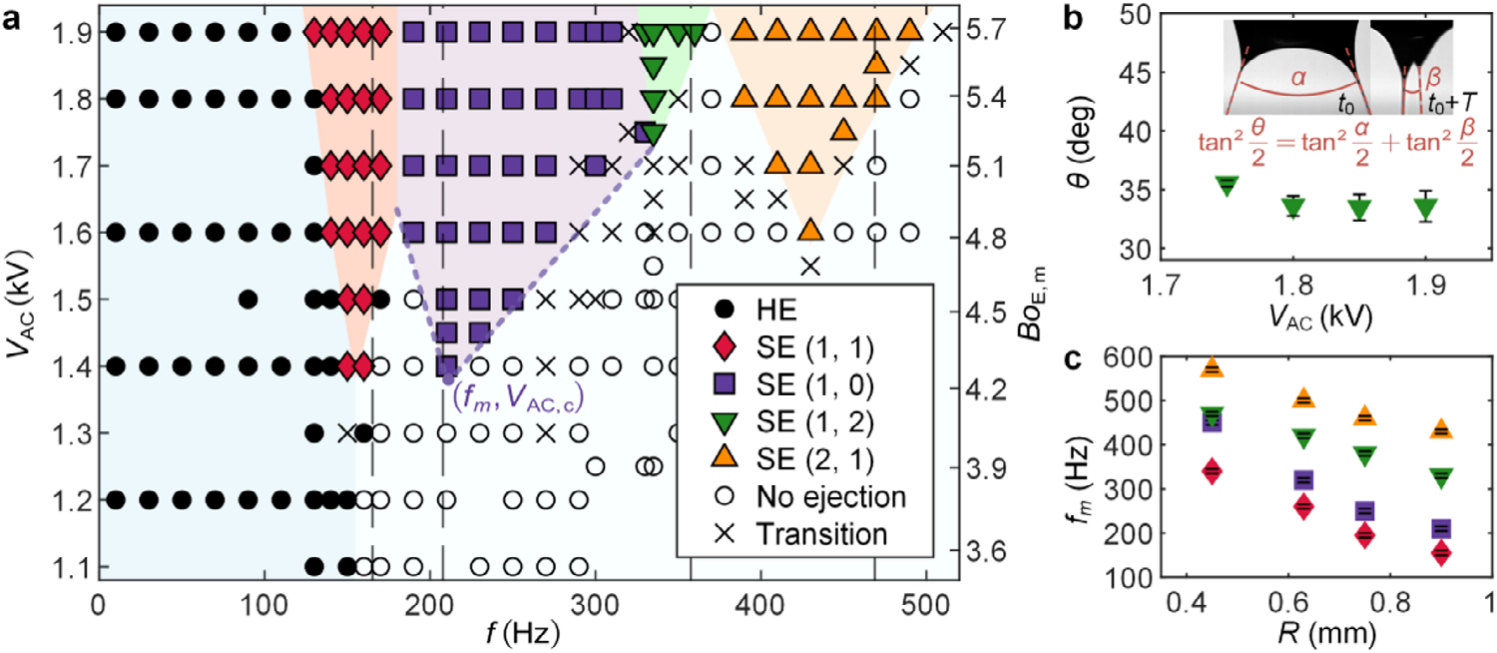
Characteristics of the dancing EHD tip streaming. a) Regime map of the dancing EHD tip streaming with respect to the frequency and the amplitude of the AC electric field. The right axis plots the maximum electric Bond number *Bo*_E,m_ = *ε*_0_ (*V*_AC_ + *V*_DC_)^2^/*Rγ*. The subharmonic ejection (SE) mode occurs at a V‐shape region with an optimal frequency *f_m_* and a critical *V*_AC,__c_. Vertical dashed lines are twice the natural frequencies Equation (3) of the meniscus obtained numerically. Here, *V*_DC_ = 1.9 kV, *Q* = 20 µL min^−1^, *R* = 0.9 mm. b) Variation of the dual‐ejection angle with applied AC voltage, corresponding to the SE (1,2) mode at *f* = 335 Hz in a). c) Optimum frequency of different ejection modes at varying nozzle outer radii. The liquid is 1‐octanol. The error bars indicate the standard deviation.

To pin down the parameter space for different modes of the dancing EHD tip streaming, we conducted systematic experiments with different oscillating electric fields. The regime map of the ejection modes with respect to *f* and *V*_AC_ is depicted in **Figure** **3**a. The modes corresponding to solid symbols were consistently reproduced in three independent runs, while the “transition” region, marked with cross symbols, denotes the parameter range where unstable or intermediate states occurred. The harmonic ejection occurs at a low *f*. As *f* increases, four subharmonic ejection modes, SE (1, 1), SE (1, 0), SE (1, 2), and SE (2, 1) are progressively excited. Higher order modes were not observed in our experiments, which may be due to the fact that the frequencies of the Faraday wave driven by the electric field are limited by the charge relaxation time *t*_*e*_ = *ε*/*σ* (≈ 1 ms), where *ε* and *σ* are respectively the dielectric coefficient and conductivity of the liquid. Regarding the angle *θ* between the two ejections, we measured *θ* under different voltages in the SE (1, 2) mode, and it is ≈35° (Figure 3b). The boundaries of these regions show completely different features compared to that of the classic harmonic ejection mode. First, the subharmonic ejections require a much higher *V*_AC_ in comparison. Second, the lower boundaries of the SE mode exhibit a V‐shape, known as the Faraday tongue,^[^ ^45^, ^47^ ^]^ indicating there exists an optimal frequency (*f_m_*) with a critical AC voltage amplitude (*V*_AC,__c_). The critical voltage amplitude increases as *f* deviates from *f_m_* for each mode. To investigate the effect of nozzle size on the ejection modes, we tested nozzles with outer radii of 0.45 mm, 0.63, 0.75, and 0.9 mm. The driving frequency was varied in steps of 10 Hz, and the optimum frequency *f_m_* of each ejection mode was determined. The uncertainty in the optimum frequency is mainly due to the step size, estimated as ±5 Hz. As shown in Figure 3c, the optimum frequency of each mode increases with decreasing nozzle size.

It is well‐known that the optimal frequency *f_m_* of the Faraday tongue is set by the natural frequency of the corresponding Faraday wave patterns.^[^ ^44^, ^45^, ^47^ ^]^ We then calculate the natural frequencies of the Faraday waves on the meniscus via numerical simulation. We consider a meniscus anchored at the nozzle exit of the same geometry parameters in an electric field, and adopt the leaky dielectric model to formulate the electrohydrodynamic governing equations at a cylindrical coordinate (*r*, *θ*, *z*) (Text S3, Supporting Information).^[^ ^39^, ^48^ ^]^ For any variable ***Φ*** representing the surface location, velocity, pressure, and electric potential, its temporal and azimuthal dependence is expressed based on the small perturbation assumption

(1) $$\Phi \left(r,\theta ,z;t\right)=\Phi_{0}\left(r,z\right)+\varepsilon \Phi^{\prime }\left(r,z\right)e^{i\omega t+im\theta }$$

where ***Φ***_0_ is the axisymmetric base flow, ***Φ*** *'* is the spatial dependence of the perturbation, *ϵ* is a small perturbation parameter, and *ω* is the eigenfrequency of meniscus oscillation. The numerical method proposed by Herrada and Montanero^[^ ^49^ ^]^ is used to solve the base flow ***Φ***_0_ and the generalized eigenvalue problem

(2) $$J_{0}\Phi^{\prime }=i\omega Q_{0}\Phi^{\prime }$$

where **J**_0_ and **Q**_0_ are Jacobians. The solution of Equation (2) (Text S4 and Figure S9, Supporting Information) gives the natural frequencies

(3) $$f_{n}=\omega_{r}/\left(2\pi t_{c}\right)$$

and the corresponding eigenmodes (*n*, *m*) of the meniscus in an electric field, where *ω_r_* (the real part of *ω*) is the dimensionless angular frequency, and *t*_*c*_ = (*ρR* ^3^/*γ*)^1/2^ with *ρ* and *γ* respectively denoting the density and surface tension of the liquid. The eigenmodes associated with the first four natural frequencies are consistent with the experimentally observed Faraday wave patterns (see insets in Figure 2a). We plot *f* = 2*f_n_* as vertical lines in Figure 3a, and found that they agree well with the optimal frequencies of the subharmonic ejection modes, with relative deviations < 10%. This demonstrates that the mode of the dancing EHD tip streaming is set by the natural frequency of the Faraday instability.

### 2.3 Threshold of the Subharmonic Ejection

**Figure 4:**
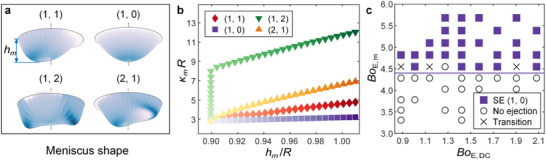
Threshold of the subharmonic ejection. a) Meniscus shape of Faraday waves with superimposed eigenmodes (1, 1), (1, 0), (1, 2), and (2, 1) obtained numerically, colored by the local surface curvature. The maximum curvature *κ_m_* occurs at the maximum height *h_m_* of the meniscus. b) Variation of *κ_m_R* with increasing *h_m_*/*R* (corresponding to increasing perturbation amplitudes in simulation). The color gradient from light to dark indicates increasing perturbation. c) Phase diagram for mode SE (1,0) regarding two electric Bond numbers, *Bo*_E,DC_ = *ε*_0_ $V_{\mathrm{DC}}^{\;2}$/*Rγ* and *Bo*_E,m_ = *ε*_0_(*V*_AC_ + *V*_DC_)^2^/*Rγ*, at *f* = 230 Hz. The solid line outlines the lower boundary of the occurrence of dancing EHD ejection. *Bo*_E,DC,_ and *Bo*_E,m_ respectively represent the DC electric effect and the maximum electric effect compared to the capillary effect.

The occurrence of the dancing EHD tip streaming also needs a sufficiently strong electric effect, characterized by the minimum voltage of the Faraday tongue in Figure 3a. For leaky dielectric fluids at an electric field, the net free charge is considered to accumulate at the interface, and the electric force acts on the surface through the Maxwell stress **τ** *_M_* = *ε* (***EE*** – *E* ^2^ **I**/2),^[^ ^50^ ^]^ where *E* ∼ *V*/*R* is the magnitude of the electric field, and **I** is the unit tensor. This electric stress not only generates the Faraday waves,^[^ ^46^ ^]^ but also overcomes capillary pressure at the wave crests, inducing local interface instability and driving tip streaming.^[^ ^6^, ^8^ ^]^ The maximum capillary pressure that must be overcome for tip growth is given by *p_c_* ∼ *γκ_m_*, where *κ_m_* denotes the peak curvature of the meniscus surface exhibiting Faraday wave patterns. Our simulation provides the shape evolution of the meniscus at different eigenmodes corresponding to the four Faraday wave patterns (**Figure** **4**a), showing that *κ_m_* occurs at the maximum height *h_m_* of the meniscus. Figure 4b plots the variation of *κ_m_R* with increasing *h_m_*/*R* (corresponding to increasing perturbation amplitudes of the eigenmodes). The inflection in the curve of mode (1, 2) marks a shift of the highest point on the meniscus, transitioning from the axis to the stagnation point on either side as the mode amplitude increases. It is obvious that mode (1, 2) manifests the maximum curvature (or capillary pressure), followed by those of modes (2, 1), (1, 1), and (1, 0) in sequence, which is consistent with the order of the critical AC voltage amplitude *V*_AC,__c_ (or electric stress) for different dancing ejection modes in Figure 3a. This indicates that the occurrence of dancing EHD tip streaming indeed comes from the local interfacial instability, i.e., the out‐of‐balance between the electric stress and capillary pressure at the Faraday wave crest.

The comparison between the electric stress (∼ *ε*_0_ *V* ^2^/*R* ^2^) and capillary pressure (∼ *γ*/*R*) yields the electric Bond number, *Bo_E_* = *ε*_0_ *V* ^2^/*Rγ*, which has been used to predict a broad range of phenomena, including the traditional EHD tip streaming, the explosion of charged droplets, and droplet coalescence induced by the electric field.^[^ ^8^, ^51^, ^52^ ^]^ In our experiments, the interfacial instability for tip streaming occurs at the peak of the oscillating voltage. We thus anticipate that the maximum voltage *V*_AC_ + *V*_DC,_ instead of *V*_AC_ sets the electric stress driving the tip streaming. This means the maximum electric Bond number

(4) $$Bo_{E,m}=\varepsilon_{0}{\left(V_{\mathrm{AC}}+V_{\mathrm{DC}}\right)}^{2}/\left(R_{\gamma }\right)$$

should determine the occurrence of dancing EHD tip streaming of different modes, as validated by our experiments with various *V*_AC_ and *V*_DC_. Compared with *Bo_E_*, the maximum electric Bond number *Bo*_E,m_ uses the peak voltage *V*_AC_ + *V*_DC_ to characterize the electric effect. It represents the ratio between the peak electric stress, *ε*_0_(*V*_AC_ + *V*_DC_)^2^/*R* ^2^, and the capillary pressure, *γ*/*R*. Taking SE (1, 0) as an example, the phase diagram in Figure 4c regarding *Bo*_E,m_ and *Bo*_E,DC_ (= *ε*_0_ $V_{\mathrm{DC}}^{\;2}$/*Rγ*) shows that a horizontal line at the critical *Bo*_E,m_ = 4.4, effectively delineates the boundary of subharmonic ejection. Similar horizontal boundary lines defined by a critical *Bo*_E,m_ are observed for other modes as well (Text S1 and Figure S5, Supporting Information). This indicates that the *Bo*_E,m_ values of the Faraday tongues in Figure 3a can extend to encompass a broader range of *V*_DC_ conditions. And the value of this critical *Bo*_E,m_ depends on the intrinsic shape of the Faraday waves at the corresponding frequency (Figure 4). Overall, these results indicate that by adjusting the oscillating voltage signal to make the *Bo*_E,m_ Equation (4), over the V‐shaped threshold curve (Figure 3a) at a frequency ≈2*f_n_* Equation (3), the dancing EHD tip streaming at different modes can be precisely controlled.

### 2.4 Applications of Dancing EHD Tip Streaming

**Figure 5:**
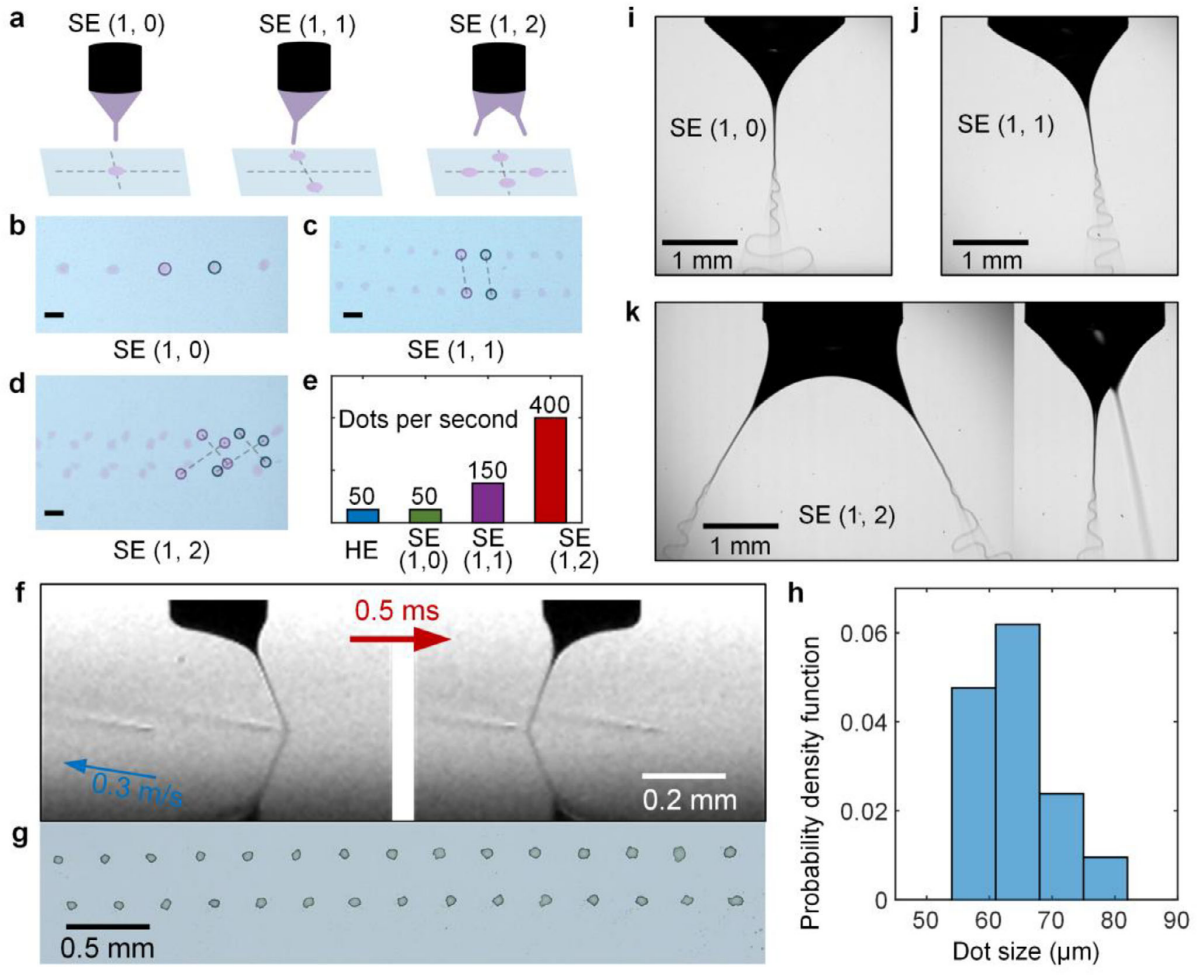
Applications of dancing EHD tip streaming in ink‐printing and electrospinning. a) Sketches illustrating the ejections in single and multiple directions. Patterns of the prints in a paper provided by different dancing EHD tip streaming modes: b) SE (1, 0) at 100 Hz, c) SE (1, 1) at 150 Hz, and d) SE (1, 2) at 200 Hz. Scale bars represent 1.0 mm. The liquid is 1‐octanol stained with Rhodamine 6G. Here, *R* = 0.9 mm, *V*_DC_ = 2.0 kV, *V*_AC_ = 1.6 kV, *Q* = 20 µL min^−1^. The purple and blue circles mark the prints from adjacent periods. e) Number of dots printed per second. Printing experiment with silver nanoparticle ink: f) high‐speed camera images of two successive ejections, g) microscopic image of the dot array printed on the glass substrate, and h) probability density function of the measured dot size. Operating conditions: nozzle outer radius *R* = 0.12 mm, *V*_DC_ = 1.2 kV, *V*_AC_ = 0.6 kV, *f* = 2000 Hz, *Q* = 0.1 µL min^−1^, substrate translation speed *U* = 0.3 m/s, ejection mode is SE (1, 0). Multi‐directional electrospinning facilitated by different subharmonic modes of 2 wt% PVB solution in ethanol: i) SE (1, 0) at 120 Hz, j) SE (1, 1) at 130 Hz, and k) SE (1, 2) at 230 Hz. Here, *R* = 0.9 mm, *V*_DC_ = 2.6 kV, *V*_AC_ = 1.4 kV, *Q* = 20 µL min^−1^.

The dancing EHD tip streaming reported here exhibits controllable, robust, and diverse ejection modes, providing a rich array of modulation options for drop‐on‐demand applications. It is well‐known that the printing speed of the traditional EHD printing is significantly constrained by the jetting frequency. In traditional EHD printing, for effective frequency control, the jetting frequency must either equal the applied voltage frequency or maintain a simple linear relationship with it. However, there exists a frequency limit, above which the jetting becomes unstable and no longer matches the driving frequency.^[^ ^33^, ^36^, ^53^ ^]^ Here we show that the high‐frequency dancing ejections modulated by Faraday instability can be utilized to increase the printing frequency and achieve special printing patterns (**Figure** **5**a). We conducted a proof‐of‐concept experiment for application in EHD printing, using 1‐octanol stained with Rhodamine 6G as ink. The grounded electrode attached to a piece of paper is fixed near the edge of a rotating motorized stage, achieving controllable movement relative to the fixed nozzle above it. Our dancing EHD tip streaming enables various patterns printed onto the paper, yielding single‐point, two‐point, and four‐point dot arrays in ejection modes of SE (1, 0), SE (1, 1), and SE (1, 2), respectively (Figure 5b–d). Notably, owing to more jet numbers per period and higher frequency, the dancing ejection modes can produce much more dots per second compared to the classic harmonic ejection mode, and the printing speed is increased by 7 times in SE (1, 2) mode (Figure 5e). We further conducted printing tests using a smaller nozzle (outer radius *R* = 0.12 mm) with a 15% solid‐content silver nanoparticle ink. A 0.1 mm thick glass slide was placed on the grounded electrode plate as the substrate, with a nozzle‐to‐substrate distance of *H* = 0.3 mm. Under a voltage frequency of 2000 Hz, dots with diameters of several tens of microns were produced at a printing frequency of 2000 Hz (Figure 5f–h; Movie S6, Supporting Information). The ejection frequency range of 10–2000 Hz achieved in this study makes it applicable to a wide variety of scenarios.

In addition to improving throughput rate, alternating ejection in multi‐directions of our dancing EHD tip streaming also allows for ejecta dispersion and multi‐path delivery. In fields such as cell and drug delivery, microfluidic systems are commonly employed for the dispersion and delivery of droplets.^[^ ^54^, ^55^, ^56^ ^]^ However, these systems face challenges such as structural complexity, low productivity, and limited scalability.^[^ ^57^, ^58^ ^]^ The inherent multi‐directional alternating delivery and high frequency characteristics make the dancing EHD tip streaming a promising candidate to solve these issues. Besides, the multi‐directional alternating ejection can also enhance the dispersion of fibers in electrospinning. As shown in Figure 5i–k, we tested 2 wt.% polyvinyl butyral (PVB) solution in ethanol, resulting in a series of new electrospinning configurations. The whipping fibers produced by single‐direction pulsating and multi‐direction alternating jets demonstrate the feasibility of applying Faraday instability‐modulated electrospray to complex fluids in material and biological manufacturing. Thus, the Faraday‐instability‐induced dancing EHD tip streaming enables grouped ejecta delivery to different target areas, potentially benefiting applications such as cell encapsulation, drug delivery, and electrospinning.^[^ ^17^, ^18^, ^54^ ^]^ Overall, the dancing tip streaming achieves multi‐directional, high‐frequency ejections from a single nozzle, offering precise temporal coordination without external synchronization, while avoiding the structural complexity and nozzle crosstalk issues inherent to multi‐nozzle strategies.^[^ ^21^ ^]^ In addition, our insights not only enable the use of dancing tip streaming modes but also help prevent them when undesired, ensuring stable operation in the conventional EHD printing.

## 3 Conclusion

We report a novel dancing EHD tip streaming phenomenon, characterized by a set of subharmonic ejection modes under high‐frequency electric fields. We unveil the mechanism behind this intriguing phenomenon as the local interfacial instability at the surface wave crests is modulated by the Faraday instability. By theoretically elucidating the parameter limits of different ejection modes in terms of frequency and amplitude of the applied voltage, we establish a control strategy for the precise modulation of ejection behavior. By implementing this strategy in EHD printing and electrospinning, we demonstrate that dancing subharmonic ejections significantly enhance droplet throughput and facilitate multi‐path delivery, surpassing the performance of classic harmonic ejection. Furthermore, our findings inspire us that broader approaches beyond Faraday waves could be considered to alter or create surface stagnation points to achieve more complex dancing tip streaming behaviors.

Building on the present findings, future work could explore broader application scenarios and test a wider range of liquids, where the roles of fluid properties remain to be further clarified. Quantitative evaluation of its performance in applications such as electrospinning would also be valuable. Moreover, employing smaller nozzles to generate even finer droplets represents a promising direction to extend the applicability of the proposed method toward micro‐ and nanoscale manufacturing. We hope that this work not only advances the fundamental understanding of EHD tip streaming but also provides a framework for manipulating local instabilities through controlled global flow, thereby opening new opportunities for electrospray applications in biological, pharmaceutical, and manufacturing industries.

## 4 Experimental Section

### Materials

1‐octanol (GC, ≥99.5%) was obtained from Aladdin (Shanghai). The dye used was Rhodamine 6G (95%, Aladdin). Ethanol (AR, ≥99.7%) used in the experiments was obtained from Jing Chun (Beijing). The polymer solution used in electrospinning was a 2 wt% PVB (M.W. 25 000–4000, Macklin) solution in ethanol. Silver nanoparticle ink with 15% solid content dispersed in a 1:1 mixture of ethylene glycol and ethanol (Sigma–Aldrich, Shanghai). The properties of the working liquids were summarized in Table S1 in the Supporting Information.

### Proper Orthogonal Decomposition (POD) of Experimental Images

POD was used to process the grayscale time series of experimental images. The collected 2D grayscale images of meniscus fluctuations and ejection phenomena at time *t* have grayscale data G(x,t), and $G^{\prime }$ is G minus its temporal mean. $G^{\prime }$ is decomposed into a set of spatially orthogonal modes modulated by time coefficients $a_{k}\left(t\right)$ (Text S2, Supporting Information)

(5) $$G^{\prime }(x,t)=\sum_{k=1}^{\infty }a_{k}(t)\Psi_{k}(x)$$

where $\Psi_{k}\left(x\right)$ is the *k*‐th POD mode and $a_{k}\left(t\right)$ is the corresponding time coefficient. By applying a fast Fourier transform (FFT) to the temporal coefficients of the dominant (first) POD mode, the power spectral density (PSD) was obtained, which reveals the presence of harmonics or subharmonics.

### Model Formulation

An infinitely large grounding electrode plate was set to perpendicular to the axis of the capillary, with a distance of *H* from the outlet of the nozzle. The triple contact lines anchor perfectly to the edge of the capillary tip, with a radius *R*. The geometry parameters were set to be the same as those in experiments, i.e., *H* = 2.0 mm and *R* = 0.9 mm. The surface position of the liquid meniscus is represented as *z* = *F*(*r*,*θ*,*t*). The ambient medium was assumed to be a perfect dielectric gas of permittivity *ε*_0_ equal to the dielectric constant of vacuum. Due to the small values of the Bond number (*Bo* = *ρɡR* ^2^/*γ* = 0.26) and the small ratios of gas‐to‐liquid density and viscosity [∼*O*(10^−3^)], the effects of gravity and gas dynamics can be neglected. The leaky dielectric model was employed to establish the electrohydrodynamic governing equations that describe the behavior of the liquid meniscus under an applied electric field (Text S3 and Figure S8, Supporting Information).^[^ ^39^, ^59^, ^60^, ^61^ ^]^

## Conflict of Interest

The authors declare no conflict of interest.

## Supporting information



Supporting Information

Supplemental Movie 1

Supplemental Movie 2

Supplemental Movie 3

Supplemental Movie 4

Supplemental Movie 5

Supplemental Movie 6

## Data Availability

The data that support the findings of this study are available from the corresponding author upon reasonable request.

## References

[1] G. I. Taylor , Proc. R. Soc. Lond. A 1934, 146, 501.

[2] S. Courrech du Pont , J. Eggers , Proc. Natl. Acad. Sci. USA 2020, 117, 32238. 33288698 10.1073/pnas.2019287117 PMC7768759

[3] J. M. Montanero , Tip Streaming of Simple and Complex Fluids, Springer Nature Switzerland, Cham 2024.

[4] I. Cohen , H. Li , J. L. Hougland , M. Mrksich , S. R. Nagel , Science 2001, 292, 265. 11303097 10.1126/science.1059175

[5] I. Hayati , A. I. Bailey , T. F. Tadros , Nature 1986, 319, 41.

[6] Y.‐H. Tseng , A. Prosperetti , J. Fluid Mech. 2015, 776, 5.

[7] J. F. De , L. Mora , Annu. Rev. Fluid Mech. 2007, 39, 217.

[8] R. T. Collins , J. J. Jones , M. T. Harris , O. A. Basaran , Nat. Phys. 2008, 4, 149.

[9] A. M. Gañán‐Calvo , J. M. López‐Herrera , M. A. Herrada , A. Ramos , J. M. Montanero , J. Aerosol. Sci. 2018, 125, 32.

[10] J.‐U. Park , J. H. Lee , U. Paik , Y. Lu , J. A. Rogers , Nano Lett. 2008, 8, 4210. 19367962 10.1021/nl801832v

[11] Z. Xu , L. Wang , X. Huan , H. Lee , J. Yang , Z. Zhou , M. Chen , S. Hu , Y. Liu , S.‐P. Feng , T. Zhang , F. Xu , Z. Chu , J. T. Kim , Adv. Sci. 2022, 9, 2103598. 10.1002/advs.202103598 PMC8844569 34939368

[12] G. Zhang , W. Li , M. Yu , H. Huang , Y. Wang , Z. Han , K. Shi , L. Ma , Z. Yu , X. Zhu , Z. Peng , Y. Xu , X. Li , S. Hu , J. He , D. Li , Y. Xi , H. Lan , L. Xu , M. Tang , M. Xiao , Adv. Sci. 2023, 10, 2206264. 10.1002/advs.202206264 PMC10104649 36782337

[13] J. B. Fenn , M. Mann , C. K. Meng , S. F. Wong , C. M. Whitehouse , Science 1989, 246, 64. 2675315 10.1126/science.2675315

[14] I. Jardine , Nature 1990, 345, 747.

[15] G. R. D. Prabhu , E. R. Williams , M. Wilm , P. L. Urban , Nat. Rev. Methods Primers 2023, 3, 23.

[16] I. G. Loscertales , A. Barrero , I. Guerrero , R. Cortijo , M. Marquez , A. M. Gañán‐Calvo , Science 2002, 295, 1695. 11872835 10.1126/science.1067595

[17] T. J. Sill , H. A. von Recum , Biomaterials 2008, 29, 1989. 18281090 10.1016/j.biomaterials.2008.01.011

[18] D. Ji , Y. Lin , X. Guo , B. Ramasubramanian , R. Wang , N. Radacsi , R. Jose , X. Qin , S. Ramakrishna , Nat. Rev. Methods Primers 2024, 4, 1.

[19] X. Cheng , Y.‐T. Liu , Y. Si , J. Yu , B. Ding , Nat. Commun. 2022, 13, 2637. 35552405 10.1038/s41467-022-30435-z PMC9098874

[20] M. R. Chowdhury , J. Steffes , B. D. Huey , J. R. McCutcheon , Science 2018, 361, 682. 30115806 10.1126/science.aar2122

[21] N. Mkhize , H. Bhaskaran , Small Sci. 2022, 2, 2100073. 40213534 10.1002/smsc.202100073 PMC11935840

[22] J. Luo , Z. Ren , X. Qi , Q. Pan , D. Li , Y. Xiong , J. Yao , H. Liu , S. Yu , J. Wei , Adv. Sci. 2025, 12, 2414122. 10.1002/advs.202414122 PMC12140340 40167187

[23] M. Cloupeau , B. Prunet‐Foch , J. Aerosol. Sci. 1994, 25, 1021.

[24] A. Lee , H. Jin , H.‐W. Dang , K.‐H. Choi , K. H. Ahn , Langmuir 2013, 29, 13630. 24102618 10.1021/la403111m

[25] R. T. Collins , M. T. Harris , O. A. Basaran , J. Fluid Mech. 2007, 588, 75.

[26] A. M. Gañán‐Calvo , J. Fluid Mech. 2004, 507, 203.

[27] M. S. Onses , E. Sutanto , P. M. Ferreira , A. G. Alleyne , J. A. Rogers , Small 2015, 11, 4237. 26122917 10.1002/smll.201500593

[28] H. Chen , J. Chen , J. Jiang , Z. Shao , G. Kang , X. Wang , W. Li , Y. Liu , G. Zheng , Sci. Rep. 2023, 13, 3790. 36882512 10.1038/s41598-023-30956-7 PMC9992658

[29] Y.u Jiang , D. Ye , A. Li , B.o Zhang , W. Han , X. Niu , M. Zeng , L. Guo , G. Zhang , Z. Yin , Y. Huang , Proc. Natl. Acad. Sci. USA 2024, 121, 2402135121. 10.1073/pnas.2402135121 PMC11145272 38771869

[30] Z. Yin , D. Wang , Y. Guo , Z. Zhao , L. Li , W. Chen , Y. Duan , InfoMat 2024, 6, 12505.

[31] Z. Esa , M. Abid , J. H. Zaini , B. Aissa , M. M. Nauman , Appl. Phys. A 2022, 128, 780.

[32] J. H. Moon , G.‐R. Yi , S.‐M. Yang , D. J. Pine , S. B. Park , Adv. Mater. 2004, 16, 605.

[33] S. Mishra , K. L. Barton , A. G. Alleyne , P. M. Ferreira , J. A. Rogers , J. Micromech. Microeng. 2010, 20, 095026.

[34] T. A. Cohen , D. Sharp , K. T. Kluherz , Y. Chen , C. Munley , R. T. Anderson , C. J. Swanson , J. J. De Yoreo , C. K. Luscombe , A. Majumdar , D. R. Gamelin , J. D. Mackenzie , Nano Lett. 2022, 22, 5681. 35819950 10.1021/acs.nanolett.2c00473

[35] L. Y. Yeo , D. Lastochkin , S.‐C. Wang , H.‐C. Chang , Phys. Rev. Lett. 2004, 92, 133902. 15089614 10.1103/PhysRevLett.92.133902

[36] L. Xu , X. Wang , T. Lei , D. Sun , L. Lin , Langmuir 2011, 27, 6541. 21506585 10.1021/la201107j

[37] J. Cheng , L. Yang , Q. Fu , J. Ren , H. Tang , D. Sun , X. Sun , Phys. Fluids 2022, 34, 012007.

[38] J.‐U. Park , M. Hardy , S. J. Kang , K. Barton , K. Adair , D. K. Mukhopadhyay , C. Y. Lee , M. S. Strano , A. G. Alleyne , J. G. Georgiadis , P. M. Ferreira , J. A. Rogers , Nat. Mater. 2007, 6, 782. 17676047 10.1038/nmat1974

[39] A. Ponce‐Torres , N. Rebollo‐Muñoz , M. A. Herrada , A. M. Gañán‐Calvo , J. M. Montanero , J. Fluid Mech. 2018, 857, 142.

[40] Y. Guan , S. Wu , M. Wang , Y.u Tian , C. Yu , W. Lai , Y. Huang , Phys. Fluids 2022, 34, 012001.

[41] K. Kim , B. U. Lee , G. B. Hwang , J. H. Lee , S. Kim , Anal. Chem. 2010, 82, 2109. 20143835 10.1021/ac9027966

[42] L. Sirovich , Q. Appl. Math. 1987, 45, 561.

[43] K. E. Meyer , J. M. Pedersen , O. Özcan , J. Fluid Mech. 2007, 583, 199.

[44] J. Miles , D. Henderson , Annu. Rev. Fluid Mech. 1990, 22, 143.

[45] X. Shao , P. Wilson , J. R. Saylor , J. B. Bostwick , J. Fluid Mech. 2021, 915.

[46] K. Ward , S. Matsumoto , R. Narayanan , J. Fluid Mech. 2019, 862, 696.

[47] A. Bongarzone , F. Viola , S. Camarri , F. Gallaire , J. Fluid Mech. 2022, 947, A24.

[48] M. A. Herrada , J. M. López‐Herrera , A. M. Gañán‐Calvo , E. J. Vega , J. M. Montanero , S. Popinet , Phys. Rev. E 2012, 86, 026305. 10.1103/PhysRevE.86.026305 23005852

[49] M. A. Herrada , J. M. Montanero , J. Comput. Phys. 2016, 306, 137.

[50] A. Castellanos , Electrohydrodynamics, Springer, Vienna 1998.

[51] J. Beroz , A. J. Hart , J. W. M. Bush , Phys. Rev. Lett. 2019, 122, 244501. 31322400 10.1103/PhysRevLett.122.244501

[52] G. Chen , P. Tan , S. Chen , J. Huang , W. Wen , L. Xu , Phys. Rev. Lett. 2013, 110, 064502. 23432252 10.1103/PhysRevLett.110.064502

[53] H. Li , W. Yang , Y. Duan , W. Chenn , G. Zhang , O. Huang , Z. Yin , Addit. Manuf. 2022, 55, 102849.

[54] R. H. Cole , S.‐Y. Tang , C. A. Siltanen , P. Shahi , J. Q. Zhang , S. Poust , Z. J. Gartner , A. R. Abate , Proc. Natl. Acad. Sci. USA 2017, 114, 8728. 28760972 10.1073/pnas.1704020114 PMC5565430

[55] Y. Liu , G. Yang , Y. Hui , S. Ranaweera , C.‐X. Zhao , Small 2022, 18, 2106580. 10.1002/smll.202106580 35396770

[56] L. Nan , M. Y. A. Lai , M. Y. H. Tang , Y. K. Chan , L. L. M. Poon , H. C. Shum , Small 2020, 16, 1902889. 10.1002/smll.201902889 31448532

[57] A. C. Daly , L. Riley , T. Segura , J. A. Burdick , Nat. Rev. Mater. 2020, 5, 20. 34123409 10.1038/s41578-019-0148-6 PMC8191408

[58] S. Damiati , U. B. Kompella , S. A. Damiati , R. Kodzius , Genes 2018, 9, 103. 29462948 10.3390/genes9020103 PMC5852599

[59] D. A. Saville , Annu. Rev. Fluid Mech. 1997, 29, 27.

[60] J. R. Melcher , G. I. Taylor , Annu. Rev. Fluid Mech. 1969, 1, 111.

[61] G. Tomar , D. Gerlach , G. Biswas , N. Alleborn , A. Sharma , F. Durst , S. W. J. Welch , A. Delgado , J. Comput. Phys. 2007, 227, 1267.

