# Supplementary material for: Dancing Electrohydrodynamic Tip Streaming Modulated by Faraday Instability
Source: Adv Sci (Weinh). 2025 Oct 14;13(2):e08649. doi: 10.1002/advs.202508649 (PMC12786353; doi:10.1002/advs.202508649)
Supplement: Supplementary file 1 — Supporting Information [file ADVS-13-e08649-s007.pdf]

Supporting Information

Dancing Electrohydrodynamic Tip Streaming Modulated by Faraday Instability

Qiyou Liu, Bingqiang Ji, Yafeng Zou, Dingwei Zhang, Hu Sun, Qingfei Fu*, and Lijun Yang**

Contents

S1. Experiments and materials
S2. Proper orthogonal decomposition of experimental images
S3. Governing equations in numerical simulation
S4. Numerical method and results
S5. Supplementary movies

S1. Experiments and materials

The experimental system shown in Supplementary Figure S1 was built, with the core components being a vertical metal nozzle and a horizontal grounded electrode. The outer radius of the nozzle is R. The injection pump provides a stable liquid flow rate of Q. The metal nozzle is connected to the output terminal of the electrical excitation system, and the voltage signal is generated by a waveform generator (UTG2062A) and amplified by a voltage amplifier (Trek 609E-6), which can generate voltage signals in the range of -4-4kV. The electrode is grounded at a distance of H from the tip of the nozzle. The visualization system includes a high-speed camera (Photron, Fastcam Nova S16), and a LED light source triggered by the camera, which are used to capture the oscillation of the meniscus and the electrospray. The high-speed camera images the electrospray at the frame rate of 4000-20000 fps, mounted with a microscope (Navitar 12X) with a magnification of 0.58-7X. In the EHD printing experiments, the grounded electrode is fixed near the edge of a motorized rotating stage, with a piece of paper attached to the electrode. By adjusting the rotation speed, the spacing between the printed dots can be controlled. A digital camera (Nikon D610) coupled with a macro lens captures the printed images from the top view.

The excitation frequency was varied from 10 to 2000 Hz, the applied voltage from 0 to 4 kV, and the flow rate from 0 to 20 μL/min. The syringe pump (LSP01-2A) has an accuracy of ≤ ±0.5%. The voltage amplifier is characterized by an output noise less than 50 mV rms and a drift with time less than 100 ppm/h. The signal generator has an amplitude accuracy of ±(1% of setting + 2 mVpp) and frequency accuracy of ±100 ppm.

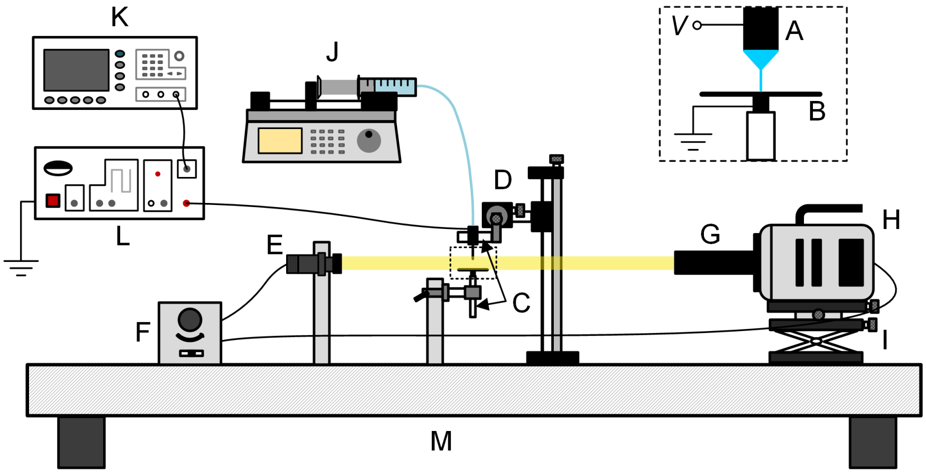

Figure S1. Schematic diagram of the experimental setup. A) nozzle, B) disk electrode, C) insulated connectors, D) four-axis displacement stage, E) LED light source, F) Pulse controller, G) Microscope, H) high-speed camera, I) triaxial displacement stage, J) syringe pump, K) waveform generator, L) high voltage amplifier, M) vibration isolation system.

1-octanol (GC, ≥99.5%) is obtained from Aladdin (Shanghai). The dye used is Rhodamine 6G (95%, Aladdin). Ethanol (AR, ≥99.7%) used in the experiments is obtained from Jing Chun (Beijing). The polymer solution used in electrospinning is a 2 wt% PVB (M.W. 25000-4000, Macklin) solution in ethanol. Silver nanoparticle ink with 15% solid content dispersed in a 1:1 mixture of ethylene glycol and ethanol (Sigma-Aldrich, Shanghai).

The physical properties of these liquids are listed in Table S1.

Table S1. Properties of the working liquids

liquid	ρ (kg/m^3)	μ (mPa·s)	γ (mN/m)	σ (S/m)	ε_r
1-octanol	827	7.5	25.0	9×10^{-8}	10.3
ethanol	789	1.1	22.3	4×10^{-6}	24.3
2 wt% PVB solution in ethanol	776	11.9	24.1	7×10^{-5}	—
silver nanoparticle ink	945	5.9	30.3	1×10^{-3}	—

By performing image analysis, we extracted the jet diameter information and compared the variations across different ejection modes, as shown in Figure S2. The results indicate that the jet diameter remains approximately 30 μm.

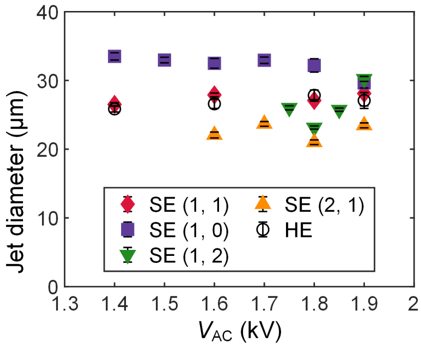

Figure S2. Comparison of jet diameters across different ejection modes. Liquid: 1-octanol. Nozzle outer radius $R = 0.9$ mm, applied DC voltage $V_{DC} = 1.9$ kV, and flow rate $Q = 20$ μL/min. The frequencies for the modes are: 110 Hz (HE), 160 Hz (SE (1, 1)), 210 Hz (SE (1, 0)), and 335 Hz (SE (1, 2)), and 450 Hz (SE (2, 1)).

To verify the long-term stability of the dancing EHD tip streaming, we conducted experiments lasting for tens of thousands of cycles, during which the ejection behavior remained highly stable. We recorded high-speed images over 200 voltage periods (*T*) (limited by the storage capacity of the high-speed camera) and overlaid them into a composite image (Figure S3). The result clearly demonstrates that the phenomenon maintains excellent periodicity and stability. The periodicity of this dancing EHD tip streaming phenomenon ensures the long-term stability when extending the running time.

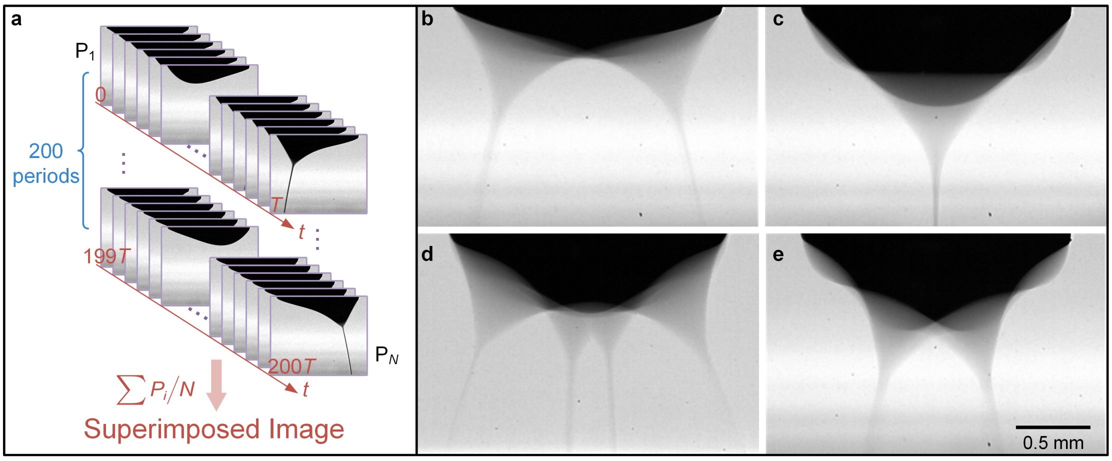

Figure S3. The long-term stability of the dancing EHD tip streaming. a) Schematic of superimposed high-speed images. Superimposed images of different dancing EHD tip streaming modes with V_{DC} = 1.9 kV, V_{AC} = 1.8 kV, R = 0.9 mm and Q = 20 μL/min at b) f = 160 Hz, c) 230 Hz, d) 335 Hz, and e) 450 Hz. Liquid: 1-octanol.

To validate that the subharmonic ejections are modulated by the Faraday wave oscillations, we observed the meniscus oscillation without ejection by cutting off the liquid supply (Q = 0). As shown in Figure S4, stable surface waves at the meniscus like the Faraday waves at a planar liquid surface are observed. Compared with the dancing EHD tip streaming phenomena under the same control parameters, the meniscus oscillations show the same modes.

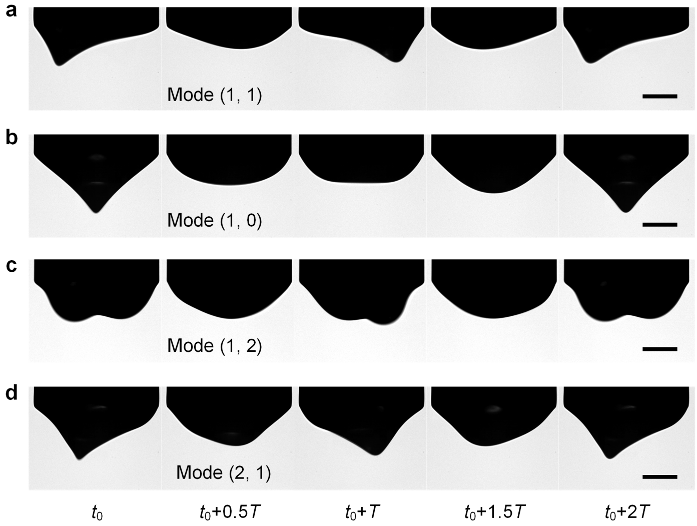

Figure S4. Faraday waves at the meniscus. Snapshots of the Faraday waves at the meniscus obtained by cutting off the liquid supply (Q = 0), with all other parameters remain consistent with those in Figure 1. The scale bars represent 0.5 mm.

We conducted experiments by varying V_{AC} and V_{DC}, with the results for SE(1, 0) shown in Figure S5a. Replot the experimental results in in a dimensionless coordinate, we find that a horizontal line of $Bo_{E,m}$ = 4.4 well predicts the occurrence of ejection (Figure S5b). Similarly, using a constant $Bo_{E,m}$ can capture the thresholds of the harmonic ejections in other modes over wide ranges of V_{AC} and V_{DC} (Figure S5c, d). Therefore, the maximum electric Bond number $Bo_{E,m}$, which compares electric and capillary effects, effectively determines the voltage threshold for dancing EHD tip streaming.

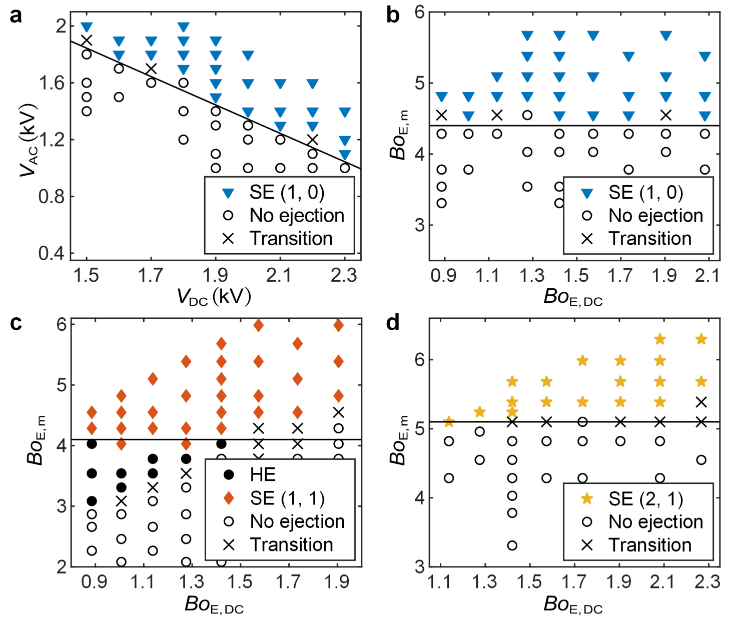

Figure S5. Voltage threshold of the dancing EHD tip streaming. a) Phase diagram for the subharmonic ejection mode SE (1,0) regarding V_{DC} and V_{AC} at a fixed voltage frequency f = 230 Hz. The solid line indicates the lower boundary of successful ejection. Phase diagrams as functions of two electric Bond numbers ($Bo_{E,m}$ and $Bo_{E,DC}$) for different ejection modes: b) SE (1,0) at f = 230 Hz, c) SE (1,1) at f = 160 Hz and d) SE (1,1) at f = 450 Hz, showing that the subharmonic ejection is set by the maximum electric Bond number, $Bo_{E,m} = \varepsilon_0 \left(V_{AC} + V_{DC}\right)^2 / R\gamma$. $Bo_{E,DC}$ = $\varepsilon_0 V_{DC}^2 / R\gamma$ representing the DC electric Bond number.

Figure S6 illustrates the dancing EHD tip streaming phenomena in ethanol, using a nozzle with an outer radius of 0.45 mm. The nozzle is positioned 3.0 mm from the grounded electrode.

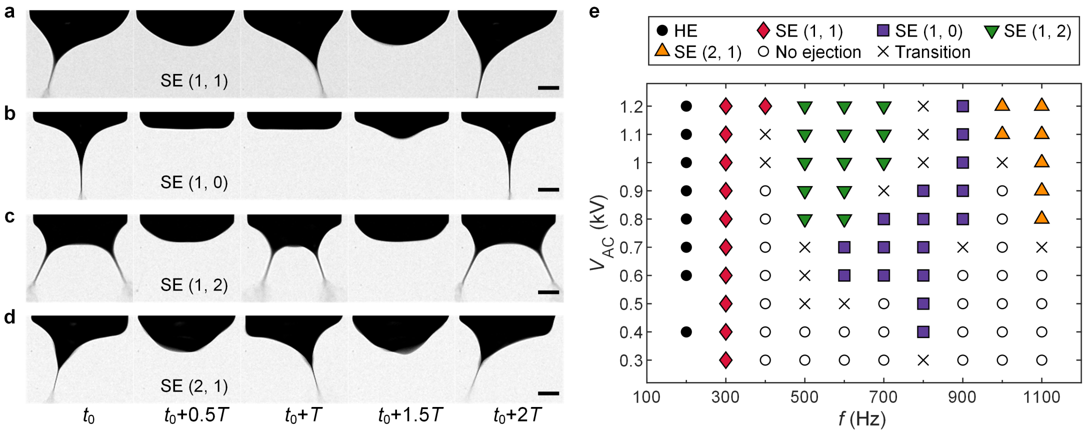

Figure S6. Different modes of the dancing EHD tip streaming in ethanol. Experimental snapshots of different subharmonic ejection (SE) modes in ethanol over 2T, with V_{DC} = 2.6 kV, V_{AC} = 1.1 kV and Q = 10 μL/min: a) SE (1, 1) at f = 300 Hz, b) SE (1, 0) at f = 900 Hz, c) SE (1, 2) at f = 700 Hz and d) SE (2, 1) at f = 1000 Hz. The nozzle with an outer radius R = 0.45 mm, is positioned 3.0 mm from the grounded electrode. The scale bars represent 0.2 mm. e) Regime map of the dancing EHD tip streaming in ethanol, with V_{DC} = 2.6 kV, Q = 10 μL/min and R = 0.45 mm.

Besides, the multi-directional ejection can also enhance the dispersion of fibers in electrospinning. As shown in Figure S7, we tested 2 wt% polyvinyl butyral (PVB) solution in ethanol, resulting in a series of new electrospinning configurations. The whipping fibers produced by single-direction pulsating and multi-direction alternating jets demonstrate the feasibility of applying Faraday instability-modulated EHD tip streaming to complex fluids in material and biology manufacturing.

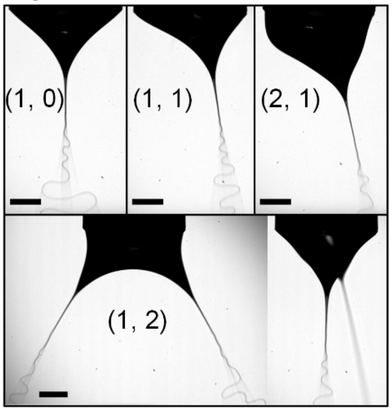

Figure S7. Multi-directional electrospinning. Multi-directional electro-spinning facilitated by different subharmonic modes: SE (1, 0), SE (1, 1), SE (1, 2) and SE (2, 1). The nozzle with an outer radius of 0.9 mm, is positioned 4.0 mm from the grounded electrode. The scale bars represent 0.5 mm.

S2. Proper orthogonal decomposition of experimental images

Proper orthogonal decomposition (POD) can be used to extract a simplified model that captures the basic characteristics and obtains the main features of flow. To measure the frequency of meniscus fluctuations and identify different modes from a series of experimental images, POD is used to process the grayscale time series of experimental images. The collected two-dimensional grayscale images of meniscus fluctuations and jetting phenomena at continuous time t have grayscale data $\mathcal{G}(\boldsymbol{x},t)$, and $\mathcal{G}'$ is $\mathcal{G}$ minus its temporal mean. $\mathcal{G}'$ is decomposed into a set of spatially orthogonal modes modulated by time coefficients

$$\mathcal{G}'(\boldsymbol{x},t)=\sum_{k=1}^{\infty}a_k(t)\Psi_k(\boldsymbol{x}), \tag{S1}$$

where the $\Psi_k(\boldsymbol{x})$ is the k-th POD mode and the $a_k(t)$ is the corresponding time coefficient.

The analysis in this paper utilizes the snapshot POD proposed by Sirovich.[1] Take a set of experimental images captured at N time points and M pixel positions as an example to illustrate the calculation process. More detailed process can be found elsewhere.[2–4] The fluctuating grayscale values are arranged in the $M \times N$ matrix

$$\mathbf{G}=\left[\boldsymbol{g}^1,\cdots,\boldsymbol{g}^N\right]=\begin{bmatrix} g_1^1 & \cdots & g_1^N \\ \vdots & \ddots & \vdots \\ g_M^1 & \cdots & g_M^N \end{bmatrix}. \tag{S2}$$

The covariance matrix is created as $\mathbf{C} = \mathbf{G}^{\mathrm{T}}\mathbf{G}$ and the corresponding eigenvalue problem

$$\mathbf{C}\boldsymbol{A}_i=\lambda_i\boldsymbol{A}_i \tag{S3}$$

is solved. The eigenvalues are ordered as $\lambda_1 \geq \lambda_2 \geq \ldots \geq \lambda_N$ and the POD modes are constructed as

$$\boldsymbol{\varphi}_i=\frac{\mathbf{G}\boldsymbol{A}_i}{\left\|\mathbf{G}\boldsymbol{A}_i\right\|}. \tag{S4}$$

For a POD mode i, the corresponding temporal coefficient is obtained by projecting the fluctuating grayscale field onto this mode: $\boldsymbol{a}_i = \mathrm{G}^{\mathrm{T}}\boldsymbol{\varphi}_i$. The energy proportion of each mode is $E_i=\lambda_i\big/\sum_{i=1}^{N}\lambda_i$. The POD mode with the highest energy proportion reflects the characteristics of the meniscus fluctuations. By performing a fast Fourier transform (FFT) on the temporal coefficients of the dominant POD mode, the power spectral density (PSD) of the temporal coefficients is obtained.[4,5] The PSD can reveal the presence of harmonics or subharmonics in the mode.

S3. Governing equations in numerical simulation

We consider the configuration sketched in Figure S8, where the electric potential of the cylindrical metal nozzle is the applied voltage V. An infinitely large grounding electrode plate is perpendicular to the axis of the nozzle, with a distance of H from the outlet of the nozzle. An incompressible liquid of density ρ, dynamic viscosity μ, permittivity ε and electrical conductivity σ is filled in the nozzle. The triple contact lines anchor perfectly to the edge of the nozzle tip, with a radius R. Establish a cylindrical coordinate system (r,θ,z) and represent the surface of the liquid meniscus at the nozzle tip as $z = F(r,\theta,z)$. The base vectors of the coordinate

system are $\hat{\boldsymbol{e}}_r$, $\hat{\boldsymbol{e}}_\theta$ and $\hat{\boldsymbol{e}}_z$. This surface has a surface tension coefficient γ. The ambient medium is assumed to be a perfect dielectric gas of permittivity ε_0 equal to the dielectric constant of vacuum.[6] Due to the small values of the Bond number ($Bo = \rho g R^2/\gamma = 0.26$) and the small ratios of gas-to-liquid density and viscosity [$\sim O(10^{-3})$], the effects of gravity and gas dynamics can be neglected.

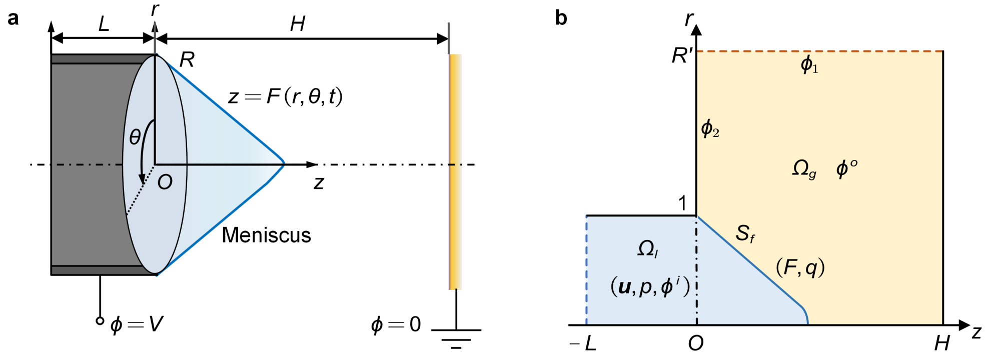

Figure S8. Physical model and computational domain in the numerical simulation. a) The physical model of the liquid meniscus is described in cylindrical coordinates (r, θ). The nozzle has an outer diameter R, and the distance between the nozzle and the grounding electrode is H. The shape of the meniscus is represented by $z = F(r, \theta, t)$, where t is the time. b) The computational domain is divided into the liquid domain Ω_l and the gas domain Ω_g, with the interface between them denoted as S_f.

All the quantities are made dimensionless with the characteristic length, time, velocity, pressure and electric field intensity as

$$R_c = R,\ t_c = \sqrt{\frac{\rho R^3}{\gamma}},\ v_c = \frac{R}{t_c},\ p_c = \frac{\gamma}{R},\ E_c = \frac{V_0}{R}. \tag{S5}$$

The resulting problem is governed by dimensionless parameters including the Ohnesorge number Oh, the electric Bond number Bo_E, the relative permittivity β and the dimensionless electrical conductivity α,

$$Oh = \frac{\mu}{\sqrt{\rho\gamma R}},\ Bo_E = \frac{\varepsilon_0 V_0^2}{R\gamma},\ \beta = \frac{\varepsilon}{\varepsilon_0},\ \alpha = \sigma\sqrt{\frac{\rho R^3}{\gamma\varepsilon_0^2}}. \tag{S6}$$

The conservation of mass and momentum equation in the liquid domain are given by

$$\nabla\cdot\boldsymbol{u} = 0, \tag{S7}$$

$$\frac{\partial\boldsymbol{u}}{\partial t} + (\boldsymbol{u}\cdot\nabla)\boldsymbol{u} = -\nabla p + Oh\nabla^2\boldsymbol{u}, \tag{S8}$$

where $u = u\hat{\boldsymbol{e}}_r + v\hat{\boldsymbol{e}}_\theta + w\hat{\boldsymbol{e}}_z$ is the velocity field and p is the pressure field in the liquid domain. Taking the superscript i to represent the inner liquid and o to represent the outer gas, the electric potential ϕ^i and ϕ^o in both the inner and outer domains follow the Laplace equation

$$\nabla^2\phi^{i,o} = 0, \tag{S9}$$

and the corresponding electric fields are represented as

$$\boldsymbol{E}^{i,o} = -\nabla\phi^{i,o}. \tag{S10}$$

At the free surface $z = F(r,\theta,t)$, the normal and tangential unit vectors to the surface are

$$\hat{\boldsymbol{n}} = \frac{-F_r\hat{\boldsymbol{e}}_r - (F_\theta/r)\hat{\boldsymbol{e}}_\theta + \hat{\boldsymbol{e}}_z}{\left(1+F_r^2+F_\theta^2/r^2\right)^{1/2}},\ \hat{\boldsymbol{t}}_1 = \frac{\hat{\boldsymbol{e}}_r + F_r\hat{\boldsymbol{e}}_z}{\left(1+F_r^2\right)^{1/2}},\ \hat{\boldsymbol{t}}_2 = \hat{\boldsymbol{n}}\times\hat{\boldsymbol{t}}_1, \tag{S11}$$

where F_r and F_θ represent derivatives of F with respect to r and θ, respectively. Therefore, the normal electric field, tangential electric field, and surface charge density q at the free surface are calculated as

$$E_n^{i,o} = -\nabla\phi^{i,o}\cdot\hat{\boldsymbol{n}},\ E_{t1} = -\nabla\phi^i\cdot\hat{\boldsymbol{t}}_1 = -\nabla\phi^o\cdot\hat{\boldsymbol{t}}_1,\ E_{t2} = -\nabla\phi^i\cdot\hat{\boldsymbol{t}}_2 = -\nabla\phi^o\cdot\hat{\boldsymbol{t}}_2, \tag{S12}$$

$$q = E_n^o - \beta E_n^i. \tag{S13}$$

In addition, we impose the kinematic and dynamic boundary conditions,

$$\frac{\partial F}{\partial t} + u\frac{\partial F}{\partial r} + \frac{v}{r}\frac{\partial F}{\partial\theta} - w = 0, \tag{S14}$$

and impose the balance of normal and tangential stresses

$$-\hat{\boldsymbol{n}}\cdot Oh\left(\nabla\boldsymbol{u} + (\nabla\boldsymbol{u})^{\mathrm{T}}\right)\cdot\hat{\boldsymbol{n}} + p - \frac{1}{2}Bo_E\left(E^{o2} - \beta E^{i2}\right) + Bo_E\left(E_n^{o2} - \beta E_n^{i2}\right) = \kappa, \tag{S15}$$

$$-\hat{\boldsymbol{t}}_j\cdot Oh\left(\nabla\boldsymbol{u} + (\nabla\boldsymbol{u})^T\right)\cdot\hat{\boldsymbol{n}} + Bo_E q\boldsymbol{E}^i\cdot\hat{\boldsymbol{t}}_j = 0,\ j = 1,2, \tag{S16}$$

at the free surface, where E^i and E^o are modules of vectors $\boldsymbol{E}^i$ and $\boldsymbol{E}^o$, respectively, and $\kappa = \nabla\cdot\hat{\boldsymbol{n}}$ is twice the mean curvature. Besides, there is surface charge conservation at the free interface

$$\frac{\partial q}{\partial t} + (\mathbf{I} - \boldsymbol{nn})\cdot\nabla\cdot(q\boldsymbol{u}) - \alpha E_n^i = 0. \tag{S17}$$

At the nozzle inlet at $z = -L$, the velocity is negligible due to the small flow rate; and at the sidewall $r = 1$, $z < 0$, the no-slip boundary condition is imposed. Moreover, the triple contact line is anchored at the end of the nozzle, $F(r = 1) = 0$. The regularity conditions are imposed at $r = 0$.

The analytical expressions of electric potentials ϕ_1 and ϕ_2 at boundaries are applied,[6]

$$\phi_1(r,z) = \frac{-K_v}{\ln(4H)}\ln\left\{\frac{\left(r^2+z^2\right)^{1/2}+z}{\left[r^2+(2H-z)^2\right]^{1/2}+2H-z}\right\},\ r = R', \tag{S18}$$

$$\phi_2 = 1 - \left[1 - \phi_1(R',0)\right]\ln r/\ln R',\ z = 0,\ 1 < r < R'. \tag{S19}$$

For variables $\boldsymbol{\Phi}(r,\theta,z,t) = \{u(r,\theta,z,t),\ p(r,\theta,z,t),\ \phi^i(r,\theta,z,t),\ \phi^o(r,\theta,z,t),\ F(r,\theta,t),\ q(r,\theta,t)\}^{\mathrm{T}}$, assuming their temporal and azimuthal dependence

$$\boldsymbol{\Phi}(r,\theta,z;t) = \boldsymbol{\Phi}_0(r,z) + \epsilon\hat{\boldsymbol{\Phi}}(r,z)\mathrm{e}^{\mathrm{i}\omega t + \mathrm{i}m\theta}, \tag{S20}$$

where $\boldsymbol{\Phi}_0(r,z)$ and $\hat{\boldsymbol{\Phi}}(r,z)$ stand for the axisymmetric base flow and the spatial dependence of the perturbation, respectively, while ω is the angular frequency and m is the azimuthal wavenumber.

For a given m, the spatial dependence of the perturbation $\hat{\boldsymbol{\Phi}}$ is the solution to the generalized eigenvalue problem

$$\mathbf{J}_0\hat{\boldsymbol{\Phi}} = \mathrm{i}\omega\mathbf{Q}_0\hat{\boldsymbol{\Phi}}, \tag{S21}$$

where $\mathbf{J}_0$ is the Jacobian of the system evaluated with the base flow, and the matrix $\mathbf{Q}_0$ accounts for the temporal dependence of the problem.[7]

S4. Numerical method and results

The numerical method proposed by Herrada and Montanero[7] is used to solve the base flow and the generalized eigenvalue problem. The inner liquid and outer gas domains are mapped onto two rectangular domains through non-singular mappings

$$r_1 = \eta_1,\ z_1 = f_1(\eta_1, \xi_1, \theta, t),\ [0 \le \eta_1 \le 1] \times [0 \le \xi_1 \le 1], \tag{S22}$$

$$r_2 = R'\eta_2,\ z_2 = f_2(\eta_2, \xi_2, \theta, t),\ [0 \le \eta_2 \le 1] \times [0 \le \xi_2 \le 1]. \tag{S23}$$

The shape functions f_1 and f_2 satisfy the elliptic equation and are obtained by solving together with the flow field and electric field.[8] Furthermore, additional boundary conditions for the shape functions are required:

For the liquid domain, the following conditions are applied: (i) regularity condition at $\eta_1 = 0$, (ii) $f_1 = L\xi_1 - L$ at $\eta_1 = 1$, (iii) $f_1 = -L$ at $\xi_1 = 0$, (iv) $f_1 = F$ at $\xi_1 = 1$ are applied. In addition, for the gas domain, the following conditions are applied: (i) regularity condition at $\eta_2 = 0$, (ii) $f_2 = H\xi_2$ at $\eta_2 = 1$, (iii) $f_2 = F$ at $\xi_2 = 0$ and $0 \le \eta_2 \le 1/R'$, (iv) $f_2 = 0$ at $\xi_2 = 0$ and $1/R' < \eta_2 \le 1$, (v) $f_2 = H$ at $\xi_2 = 1$ are applied.

Figure S9a illustrates the mapping and grid utilized in this study. The equations and variables are discretized in the ξ direction using 31 Chebyshev spectral collocation points in both the liquid and gas domains. However, in the η direction, we employ fourth-order finite differences with 31 and 91 equidistant points in the liquid and gas domain, respectively. After independence analysis, we confirm that the results presented in this work do not depend on the mesh and domain size. In our calculations, we used the same parameters as in the experiments, and the corresponding values of the dimensionless parameters are $R' = 3$, $L = 1$, $H = 20/9$, $Oh = 0.055$, $Bo_E = 1.421$, $\alpha = 49.916$ and $\beta = 10.3$. First, the base flow is calculated, with the interface shape of the base flow shown in Figure S9b. Building on this, the eigenvalue problem for the perturbations is solved, resulting in the eigenvalues ω and corresponding eigenvectors. The eigenvalue spectrum is presented in Figure S9c. The natural frequencies are obtained from $f_n = \omega_r/(2\pi t_c)$.

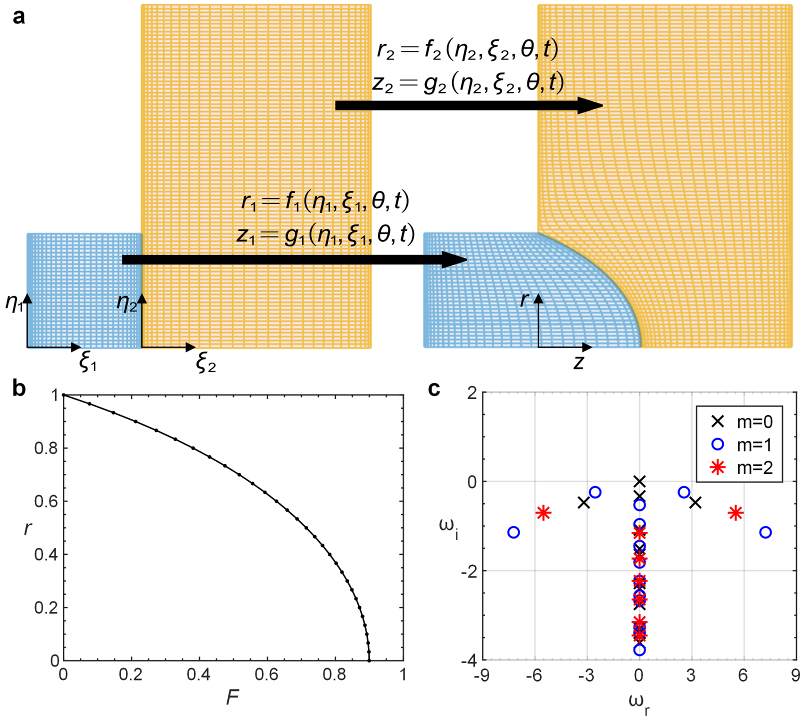

Figure S9. a) Computational subdomains and grids for the original and mapped variables. The blue lines represent the liquid mesh, and the orange lines represent the gas mesh. The left panel shows the numerical domain, while the right panel shows the physical space. b) The interface shape of the base flow. The computed interface profile $z = F(r)$ for the axisymmetric base flow, with Oh = 0.055, Bo_E = 1.421, α =49.916 and β = 10.3. c) Eigenvalue spectrum. Calculated eigenvalues ω in the complex plane for azimuthal wavenumbers m = 0, 1 and 2. The real part ω_r represents the dimensionless angular frequency, and the natural frequencies are obtained from $f_n = \omega_r/(2\pi t_c)$, where $t_c = (\rho R^3/\gamma)^{1/2}$ = 0.0049 s. For mode (1, 1), ω_r = 2.55 and f_n = 82.62 Hz; for mode (1, 0), ω_r = 3.21 and f_n = 103.90 Hz; for mode (1, 2), ω_r = 5.52 and f_n = 178.81 Hz; and for mode (2, 1), ω_r = 7.23 and f_n = 234.44 Hz.

S5. Supplementary movies

Movie S1. Experimental video of the harmonic ejection mode HE at liquid flow rate $Q = 20$ μL/min. The applied voltage frequency is $f = 90$ Hz (period $T = 11.11$ ms).

Movie S2. Experimental video of the subharmonic ejection mode SE (1, 1) at liquid flow rate $Q = 20$ μL/min and the corresponding Faraday wave mode (1, 1) at $Q = 0$. The applied voltage frequency is $f = 160$ Hz (period $T = 6.25$ ms).

Movie S3. Experimental video of the subharmonic ejection mode SE (1, 0) at liquid flow rate $Q = 20$ μL/min and the corresponding Faraday wave mode (1, 0) at $Q = 0$. The applied voltage frequency is $f = 230$ Hz (period $T = 4.35$ ms).

Movie S4. Experimental video of the subharmonic ejection mode SE (1, 2) at liquid flow rate $Q = 20$ μL/min and the corresponding Faraday wave mode (1, 2) at $Q = 0$. The applied voltage frequency is $f = 335$ Hz (period $T = 2.99$ ms).

Movie S5. Experimental video of the subharmonic ejection mode SE (2, 1) at liquid flow rate $Q = 20$ μL/min and the corresponding Faraday wave mode (2, 1) at $Q = 0$. The applied voltage frequency is $f = 450$ Hz (period $T = 2.22$ ms).

Movie S6. Experimental video of printing with silver nanoparticle ink. The applied voltage frequency is $f = 2000$ Hz.

References

[1] L. Sirovich, *Q. Appl. Math.* **1987**, *45*, 561.

[2] K. E. Meyer, J. M. Pedersen, O. Özcan, *J. Fluid Mech.* **2007**, *583*, 199.

[3] C. W. Rowley, S. T. M. Dawson, *Annu. Rev. Fluid Mech.* **2017**, *49*, 387.

[4] M. Arienti, M. C. Soteriou, *Phys. Fluids* **2009**, *21*, 112104.

[5] H. M. Ek, V. Nair, C. M. Douglas, T. C. Lieuwen, B. L. Emerson, *J. Fluid Mech.* **2022**, *930*, A14.

[6] A. Ponce-Torres, N. Rebollo-Muñoz, M. A. Herrada, A. M. Gañán-Calvo, J. M. Montanero, *J. Fluid Mech.* **2018**, *857*, 142.

[7] M. A. Herrada, J. M. Montanero, *J. Comput. Phys.* **2016**, *306*, 137.

[8] Y. Dimakopoulos, J. Tsamopoulos, *J. Comput. Phys.* **2003**, *192*, 494.
